# BRCAFem: A database for breast cancer research

**DOI:** 10.6026/973206300200473

**Published:** 2024-05-31

**Authors:** Ghazala Sultan, Swaleha Zubair

**Affiliations:** 1 Department of Computer Science, Faculty of Science, Aligarh Muslim University, Aligarh-202002, India; 2 Department of Computer Science, Faculty of Science, Aligarh Muslim University, Aligarh-202002, India

**Keywords:** breast cancer, database, sequencing datasets, imaging data, genes, drugs

## Abstract

Amid extensive breast cancer research, valuable data and findings often remain scattered across published literature, databases and web resources, posing challenges for researchers and practitioners in curating specific datasets, genes and relevant information. Hence, we developed BRCAFem (BReast CAncer of Females), an integrated database for breast cancer research. BRCAFem includes 1220 breast cancer genes, 82 FDA-approved breast cancer prevention and treatment drugs and 33 sequencing and imaging datasets. Additionally, BRCAFem provides general information about breast cancer, global statistics, risk factors, treatment options and blogs related to recent updates in breast cancer research.

## Background:

Breast Cancer (BRCA) is the most prevalent cancer affecting millions of women worldwide. Statistically, it accounts for 32% of all diagnosed cancers, which represents 1 in 4 cancers diagnosed among women, whereas it ranks as the most common cause of cancer-related fatalities in women [1]. The estimate projected by World Health Organization (WHO) for total number of new breast cancer cases is to vary from 2.26 million to 3.19 million between the years 2020 and 2040, with mortality rates expected to increase from 0.69 million to 1.04 million; this rise of 41.15% in breast cancer cases over the next two decades highlights the urgency for global efforts to enhance awareness, early detection and access to quality healthcare services [1]. It is reported that the most common cancers in females constitute 52% of all new cancer diagnoses every year, out of which BRCA alone accounts for 31% of the cases [2]. Notably, its severity is not confined to the breast tissues, but when not detected early, it worsens into invasive carcinoma and metastasizes to other organs of the body [3, 4, 5]. In addition to the psychological burden, the economic impact of breast cancer is non-negligible. The economic burden is measured based on the Cost of illness (COI), which encompasses direct costs such as diagnosis and treatment expenses, indirect costs including the value of reduced or lost productivity suffered by the patient, and intangible costs comprising the cost of pain and suffering [6]. There has been an alarming increase in national expenditures, including medical services and other cancer care, within a decade, and it escalated from less than 90 billion in 2010 to 193 billion dollars in 2020, where breast cancer has the highest treatment cost with an estimated economic burden of 29.8 billion dollars [7]. The recent statistic indicates the urgency of rigorous research in order to control the perpetually growing incidence rate of breast cancer cases. It is important to note that databases and other web repositories have significant contributions to research studies as they hold relevant data for analysis and insights corresponding to previous research studies [8, 9]. There exist various databases that assist researchers in understanding breast cancer based on risk factors, symptoms, diagnosis, and treatment options; some of them summarize genes, proteins and drug associativity studies [10, 11]. Therefore, it is of interest to develop an extensive database with data, information, updates, statistics, genes, proteins and drugs together within a single repository to provide an end-to-end platform for breast cancer research in order to minimize compromised data accessibility and time intricacies.

## Materials and Methods:

**Figure 1:**
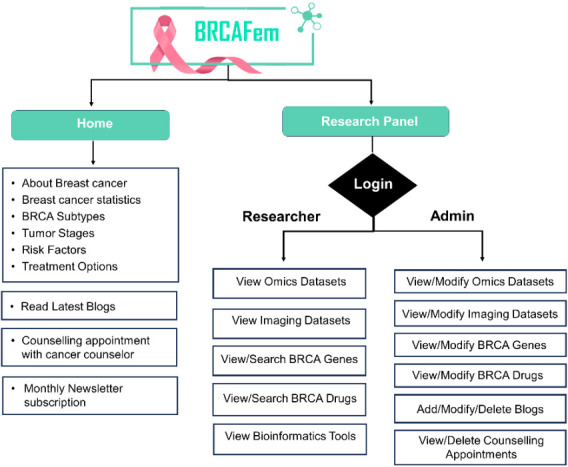
Schematic diagram representing BRCAFem.

The primary objective of the database is to provide a complete solution to breast cancer researchers seeking related data. Figure 1 outlines BRCAFem sections, data availability within the database and users' accessibility to the data. BRCAFem is broadly divided into two main sections: the Home Page and the Research Panel. The Home Page provides general information about the database and breast cancer, while the Research Panel is designed to meet the requirements of breast cancer researchers, offering targeted data and relevant information.

**Figure 2:**
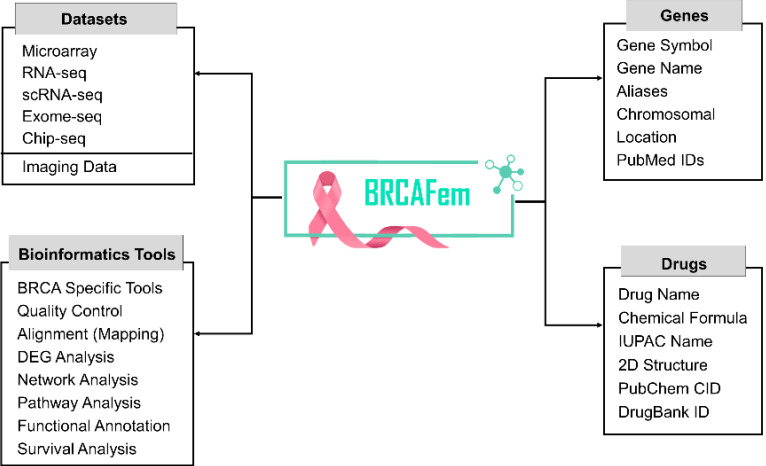
Overview of data in the Research panel of BRCAFem.

Furthermore, the breast cancer-related existing datasets, reported genes, and approved drug molecules that were scattered over discrete web repositories were thoroughly curated and categorically presented within BRCAFem database, as shown in Figure 2. In this version of BRCAFem, we have included breast cancer datasets retrieved from NCBI, 10xGenomics and other repositories using keywords "Breast AND Homo Sapiens", "Breast Cancer", "DCIS", "IDC", "ILC", "TNBC" [12, 13]. The accession ID and sample counts were noted separately based on data type, including microarray, RNA-seq, scRNA-seq, and Exome-seq data. Furthermore, the breast cancer imaging dataset includes MRI (Magnetic Resonance Imaging), CT (Computed Tomography) Scan and PET (Positron Emission Tomography) Scans retrieved from Cancer Imaging Archive. The breast cancer genes were retrieved from human disease database MalaCard, corresponding to all types of breast cancers. MalaCards provides comprehensive information on all annotated human maladies integrated from 75 different web sources and modelled on the GeneCards database [14]. The gene section is meticulously crafted, considering crucial parameters that include gene official symbol, gene name, gene aliases, chromosomal location and PubMed reference for individual genes. Similarly, breast cancer drugs approved by the FDA were retrieved from National Cancer Institute (NCI) drug repository [15, 16]. It provides drugs listed in 3 categories i.e. drugs approved to prevent breast cancer, drugs for breast cancer treatment and drug combinations widely accepted in breast cancer management.

The dynamic front-end of BRCAFem is designed using PHP, HTML and JavaScript. PHP (Programming language Hypertext Preprocessor) and HTML (HyperText Markup Language) are employed as front-end tools due to its versatility as general-purpose scripting. Especially to utilized for web application development. JavaScript is used for design and client-side validation. The crafted frontend design is backed up XAMPP server dedicated for Cross-Platform, Apache, MySQL, PHP and Perl [17]. The backend of BRCAFem is established on MySQL, a relational database management system equipped with management tools and technical support. MySQL is compatible with most operating systems and serves as a robust database management system [18].

## Results and Discussion:

## Database features:

**Figure 3:**
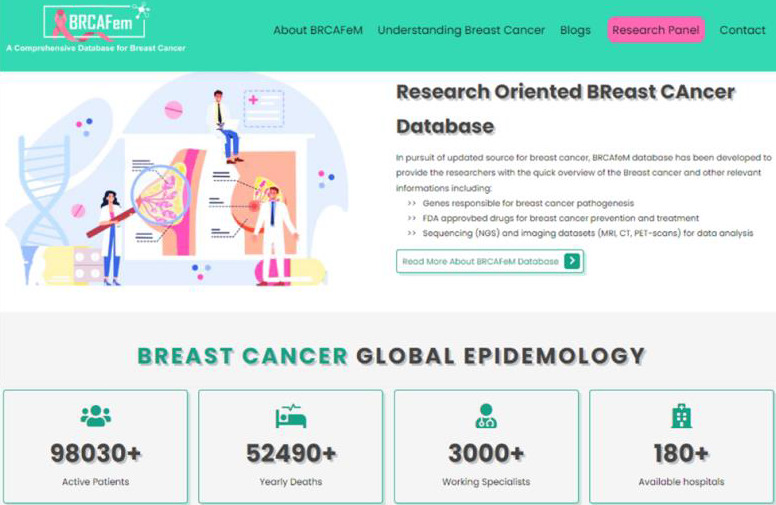
Home page of BRCAFem.

The home page includes the master navigation bar to move through different pages which includes a database description, breast cancer global statistics and related blogs (Figure 3). The epidemiology section further expands into the details of breast cancer to understand why and how it occurred, risk factors, cancer stages, screening methods and treatment options.

**Figure 4:**
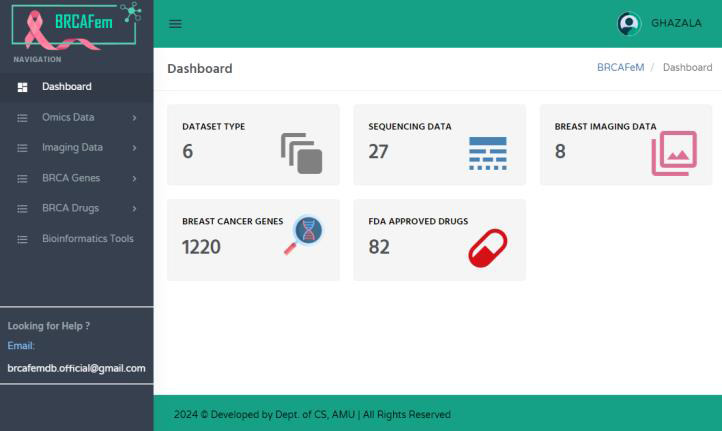
The researcher's dashboard to access datasets, genes, drugs and tools.

Furthermore, the "Research Panel" is specifically designed to serve the needs of breast cancer researchers. After successful registration and login, the database can be searchable for sequencing datasets, imaging datasets, genes and drugs through the user dashboard (Figure 4). The research panel of BRCAFem database is comprised of four components: datasets, genes, drugs and tools.

**Figure 5:**
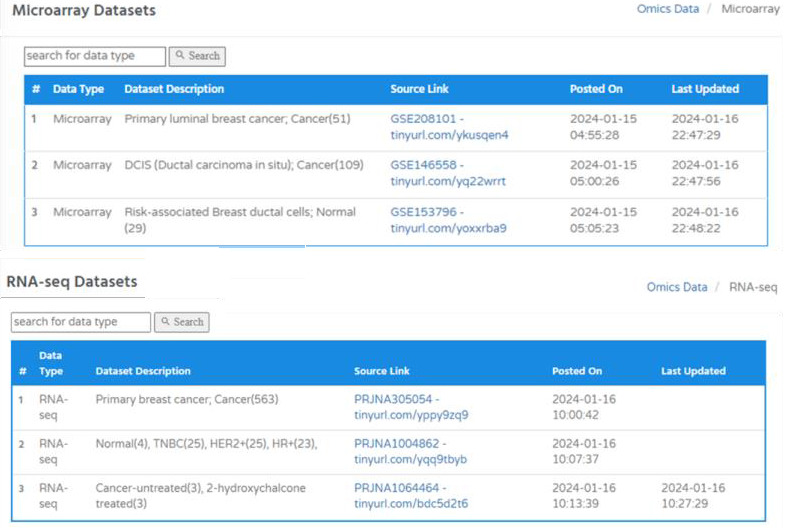
The omics (sequencing) datasets in BRCAFem

The datasets are organized categorically in a tabular format that includes data type, dataset description, source link, date on which data was uploaded, and when the dataset was last updated. These datasets could be retrieved directly through the accession ID and associated short hyperlink. These URLs will redirect the user to the primary source of the dataset (Figure 5). One of the notable features about the datasets presentation is that the user will be able to see sample conditions and number of samples within each dataset, to make it convenient while selecting the dataset for analysis.

**Figure 6:**
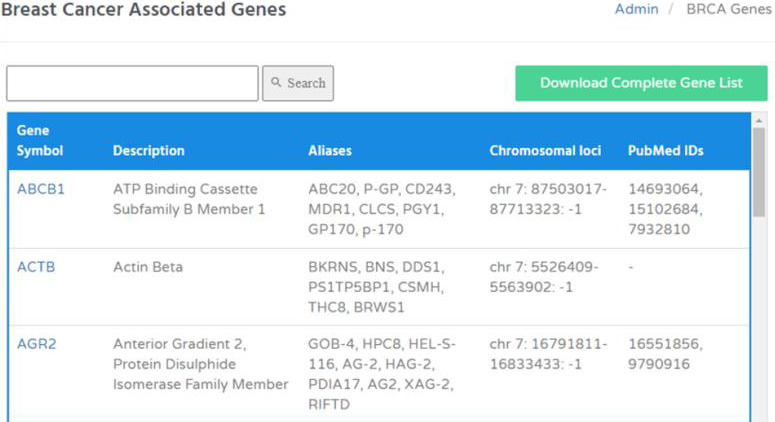
Breast Cancer Genes in BRCAFem.

The breast cancer-associated 1220 genes included within BRCAFem can be accessed through gene official symbol, gene name and chromosomal loci (chromosome number or position). The gene aliases, or alternates are also included in the table to deal with occasional name updates of various genes. Moreover, the PubMed IDs of the published articles are included corresponding to each gene to get a comprehensive understanding of the role of the genes in breast cancer pathogenesis (Figure 6). The user can also download the complete gene list in CSV (Comma Separated Values) format.

**Figure 7:**
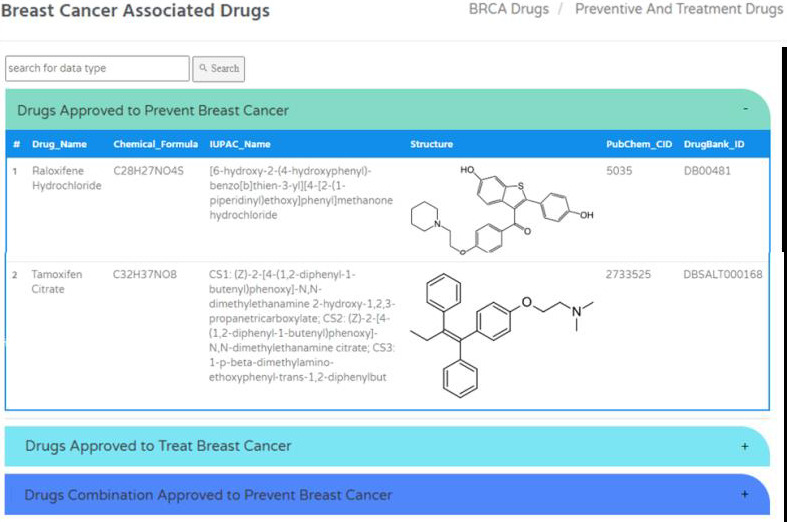
Breast Cancer Prevention and Treatment Drugs in BRCAFem

Drug table includes total 82 drugs specific to breast cancer which is created based on parameters such as drug name, chemical formula, IUPAC name, structure, PubChem CID and DrugBank ID (Figure 7). The search box enables users to find specific drugs. Similarly, tools section includes bioinformatics analysis tools for sequencing data analysis, risk analysis, survival analysis specific to breast cancer as well as general purpose tools Breast cancer research relevant active communities can also be browsed through this section.

## Future developments:

The BRCAFem database will be regularly updated, backed by user's feedback and requirements, to ensure the availability and usability of up-to-date information on breast cancer. We aim to integrate cloud storage for the datasets within BRCAFem that enable direct data download. Future updates also include the functional annotation of genes, pathway mapping, and gene-gene network integration. In addition, more genes, drugs and tools will be incorporated into the existing database, and other limitations will be improved in the forthcoming updates.

## Conclusion:

BRCAFem is a breast cancer-specific database that offers hassle-free data access for creating valuable insights. Along with general updates on breast cancer, the panel provides a separate section to access related datasets, genes, drugs and data analysis tools included in our database. The availability of sequencing datasets, imaging data, breast cancer-specific genes and drugs within one specific platform makes BRCAFem a unique database in its discipline.
